# Validation of an Instrument for Detecting Problematic Internet Use in Adolescents

**DOI:** 10.3390/bs14080715

**Published:** 2024-08-15

**Authors:** Mateo Pérez-Wiesner, Kora-Mareen Bühler, Jose Antonio López-Moreno

**Affiliations:** 1 Department of Psychobiology and Methodology in Behavioral Sciences, Faculty of Psychology, Somosaguas Campus, Complutense University of Madrid, 28223 Madrid, Spain; mateop01@ucm.es (M.P.-W.); kobuhler@ucm.es (K.-M.B.); 2 Department of Psychology, Faculty of Health Sciences—HM Hospitals, University Camilo José Cela, 28692 Madrid, Spain; 3 MIDELOY Research-Madrid, 28922 Madrid, Spain

**Keywords:** problematic internet use, internet addiction, assessment, measurement, positive emotional regulation, emotional processes

## Abstract

Problematic internet use (PIU) has drawn attention due to its potentially negative consequences on individuals’ social and personal lives. At present, a consensus on diagnostic criteria for problematic internet use remains elusive, leaving uncertainty regarding its classification as a distinct mental disorder. Extensive research efforts are underway to investigate its underlying causes, risk factors, and correlated adverse consequences. Nonetheless, research on problematic internet use (PIU) frequently faces challenges due to the absence of consistent and dependable evaluation methods, with many existing assessment tools lacking a solid theoretical basis. This study introduces a novel instrument that incorporates positive emotional regulation along with negative emotional regulation, compulsive use, and cognitive preoccupation, all crucial aspects of PIU. The study involved 3054 adolescents from Madrid, Spain, and employed exploratory and confirmatory factor analyses to validate the instrument’s structure. The resulting four-factor model includes Positive Emotional Regulation, Negative Emotional Regulation, Compulsive Use, and Cognitive Preoccupation. The instrument demonstrates good internal consistency and an association with risk factors, as evidenced by correlations with time spent on various internet-related activities. This comprehensive tool enhances our understanding of PIU and its underlying cognitive and emotional processes and provides a valuable resource for assessing and addressing problematic internet use in adolescents.

## 1. Introduction

The impact of internet use on adolescents has increased the interest in studying behaviors that lead to problematic use. This interest has led to the inclusion of Internet Gaming Disorder (IGD) in the latest diagnostic manuals, specifically in Section III of the Diagnostic and Statistical Manual of Mental Disorders (DSM-5-TR), as a recommendation for future studies with non-clinical diagnostic criteria. The ICD-11, on the other hand, has included it with specific criteria. Both manuals are based on the similarities between offline and online gambling disorder (GD).

In Davis’s initial theory (2001) [1], an initial conceptualization of problematic internet use was proposed, which, after twenty years, has been further defined and explained [2]. Currently, Problematic Internet Use (PIU) is considered the most widely used term because it is seen as a holistic term that encompasses, for example, IGD and GD included in diagnostic manuals, as well as problematic or addictive use of social networks or instant messaging [2]. According to the scientific literature, all these concepts share broad similarities, such as maladaptive behavior patterns that interfere with the individual and lead to negative social and personal consequences. The core patterns of loss of control, tolerance, and withdrawal are also shared [2,3,4,5]. The difference is that in GD, gambling can be online or offline, whereas in other cases, it is always through the internet.

Different labels have been assigned to the different conceptualizations of problematic internet use (PIU), such as internet addiction, compulsive internet use, excessive internet use, and pathological internet use, among others [2,6,7]. Despite this diversity of terms, we found common variables in the different studies, for example, excessive time dedication, uncontrolled or impulsive use, dependency, intrafamily conflicts, academic problems, etc. [2,7,8,9,10]. Therefore, PIU can be understood as a continuum between internet use and problematic use represented by a series of maladaptive behaviors of the individual that can lead to negative social and personal consequences [7,8,10,11,12,13].

Currently, there is no scientific consensus that PIU meets a diagnostic criterion for a mental disorder [8,10,14]. The lack of diagnostic criteria, underlying theories, and the need to better conceptualize PIU have led to numerous studies aimed at determining its causes, risk factors, and/or negative consequences associated with PIU [2,7,8,9,13,14]. These studies can be mainly classified into two types, those that use diagnostic or screening instruments, and those that use instruments to evaluate psychological processes, evaluating the negative consequences in both types of studies such as lack of sleep, poor academic performance, or increased time spent using the Internet [2,8,9,15,16,17]. Preferring the adolescent population due to their distinct internet usage patterns compared to adults, problematic internet use is higher among adolescents, particularly with a greater inclination towards social networks, online gaming, and consuming multimedia content [18,19]. Diagnostic/screening instruments have not been based on a theoretical model. This has been criticized by many authors, including Laconi, Rodgers, and Chabrol, 2014 [16]; and Brand, Laier, and Young, 2014 [6]. In contrast, instruments designed to determine psychological processes aim to validate the theoretical model underlying a pathology and its corresponding negative consequences [6,20].

Within the scientific literature on these latter instruments, among those designed to determine psychological processes, there are mainly six theoretical models that attempt to explain the causes of PIU [1,4,6,20,21,22]. Of these six, only two have undergone empirical validation: the Generalized Internet Addiction Model [6] and the Generalized Problematic Internet Use Model [20]. Both are based on the model proposed by Davis (2001) [1].

On the other hand, despite the existence of numerous instruments assessing PIU, none of the most widely used ones are recent creations except for the Problematic Internet Use Questionnaire-Short Form (PIUQ-SF-6) developed by Demetrovics et al. (2016) [23]. However, this is a shortened version as previous versions are older compared to other instruments like the GPIUS derived from Caplan’s 2010 [20] model. Furthermore, they are based on Young’s IAT questionnaire (1998) [17], which is not grounded in any theoretical model but purely on diagnostic criteria for GD, not IGD. The only instrument based on a theoretical model and with sufficient empirical evidence is the GPIUS2 [10,20].

The “Generalized Internet Addiction” model posits that coping style and expectations towards the internet are causes of Generalized Problematic Internet Use. The “Generalized Problematic Internet Use” model posits that compulsive use, cognitive preoccupation, mood regulation, and preference for online social interaction are the processes leading to negative consequences of internet use. Although the latter model [24] has been empirically validated in different populations worldwide, it still shows certain important limitations. On the one hand, the construct of preference for online social interaction, built on the basis of social anxiety [25], does not imply that cognitive-behavioral processes can appear in the absence of this; for example, individuals can use the Internet in a problematic way to browse for the mere fact of escaping their problems and wanting to interact with other individuals [26]. These subjects would be discriminated against by the Generalized Problematic Internet Use model [7,14]. On the other hand, the “mood regulation” construct has been designed solely based on negative emotional experience [24], but this does not allow us to identify whether positive emotional regulation plays a causal role in Generalized Problematic Internet Use. Recent studies have demonstrated the critical role of positive emotional regulation in problematic internet use and other applications, such as video games, social networks, and instant messaging [27,28].

Taking all this evidence into account, we consider it necessary to introduce the construct of positive emotional regulation into the model proposed by Caplan (2010) [20], where the construct of positive emotional regulation is understood as a tendency to maintain one’s mood through the use of the Internet in the absence of other regulation strategies [29]. To accomplish this, we have designed an instrument that incorporates positive emotional regulation, along with negative emotional regulation, compulsive use, and cognitive preoccupation. An instrument tailored for adolescents can address these specific dimensions of internet use, providing a more comprehensive and accurate assessment. This would help identify those at risk and implement early and appropriate interventions [18,19]. Our data effectively confirm that positive regulation plays a determining role in the psychological processes that explain Generalized Problematic Internet Use.

## 2. Materials and Methods

Participants

A total of 3891 adolescents from various schools in the Community of Madrid who were enrolled in Obligatory Secondary Education (Educación Secundaria Obligatoria, ESO) courses participated in the study. After applying the inclusion criteria—which included being enrolled in an ESO course, not having a diagnosed mental disorder, holding Spanish nationality, and completing all the proposed items—837 participants were excluded, resulting in a final sample of 3054 participants. Among these, 1527 were male (M = 13.56 ages, SD = 1.26) and 1527 were female (M = 13.54 ages, SD = 1.22). Based on their academic year, 22.6% (*n* = 689) were in 1st year of ESO, 28.1% (*n* = 857) were in 2nd year, 28.2% (*n* = 860) were in 3rd year, and 21.1% (*n* = 648) were in 4th year.

Variables and Instruments

For the assessment of maladaptive behavior towards information and communication technologies, an ad hoc questionnaire was developed and administered (Supplementary Materials S1) referred to as the MBIN (Maladaptive Behavior on the Internet) questionnaire. This questionnaire consisted of 19 items designed to operationalize positive mood regulation, negative mood regulation, compulsive use, and cognitive preoccupation. In formulating the statements for positive and negative mood regulation and for the lack of valence in models of problematic Internet use, reference from Whiteside and Lynam’s (2001) [30] model of the UPPS Impulsive Behavior Scale (adapted by Verdejo-García et al., 2010 [31]) for substance addictions was drawn. The factors of positive and negative urgency from this model have shown scientific evidence in the context of maladaptive behavior toward information and communication technologies [32]. Specifically, negative urgency has been related to the use of the Internet as a coping mechanism for negative emotional states, while positive urgency has been related as a mechanism of immediate reward, in both cases due to a lack of coping strategies [10,32,33].

The statements for the cognitive preoccupation factor were based on Meyer, Miller, Metzger, and Borkovec’s (1990) [34] model, specifically the Penn State Worry Questionnaire (Spanish adaptation by Sandín, Chorot, Valiente, and Lostao, 2009 [29]) that has shown scientific results in the prediction and association with problematic internet use [10,35]. The statements of cognitive preoccupation were phrased in reverse to prevent acquiescence bias. The remaining statements were based on the theoretical model proposed by Caplan (2010) [20] in the Generalized and Problematic Internet Use Scale (GPIUS2) questionnaire, which had been validated in the Spanish population by Gámez-Guadix (2013) [36].

The response options for the statements consisted of a Likert-type scale, where 1 corresponded to “completely disagree”, and 6 corresponded to “completely agree”, to avoid central tendency [11]. In addition to the questionnaire, participants were asked to evaluate the time they spent using different information and communication technologies, such as browsing the internet, social media, and instant messaging, as an external variable to assess criterion validity. Participants had to indicate the number of hours spent on the internet from Monday to Friday and from Saturday to Sunday, within a range of 0 h to more than 10 h of use.

Study procedure

Fifty school centers in the Community of Madrid were contacted, of which Eighteen chose to participate. All students were informed through the school and the association of parents. After receiving the informed consents and assents signed by the guardians and the participants, the questionnaires were administered in digital format via tablets during school hours and in the presence of tutors from different courses, as well as in the presence of a psychologist throughout the 2022–2023 academic year. Participation was voluntary and anonymous. The project received approval from the Ethics Committee of the Complutense University of Madrid.

Data analysis

Once the data were collected, descriptive analyses of the sample were conducted, segmented by academic year. To determine the number and composition of factors, an exploratory factor analysis (EFA) using Maximum Likelihood estimation with Varimax rotation and without specifying the number of factors was performed [37]. Confirmatory factor analysis (CFA) was employed to assess the factorial validity of the model proposed by the EFA. Model fit was evaluated using goodness-of-fit statistics, including Chi-square (X2), the ratio of Chi-square to degrees of freedom (where 5.0 indicates a good model fit), Normed Fit Index (NFI), Comparative Fit Index (CFI), Root Mean Square Error of Approximation (RMSEA), and Standardized Root Mean-Square Residual (SRMR). A well-fitting model typically exhibits CFI and NFI values ≥ 0.95, RMSEA ≤ 0.05, and SRMR < 0.05 [26,38].

The internal consistency of the items was determined using Cronbach’s alpha statistic. Finally, to verify the relationship of the factors resulting from the CFA with risk variables, such as time dedicated to different information and communication technologies during the week and on weekends and academic performance, the Pearson correlation coefficient was calculated.

All analyses were conducted using SPSS version 25 for Windows and AMOS 26 for Windows software.

## 3. Results

The suitability of the sample for analysis was verified using the Kaiser–Meyer–Olkin (KMO) statistic, which yielded a value of 0.9, indicating its adequacy for factorization [13]. Table 1 displays the factor loadings of each item, with items having loadings below 0.40 and those loading onto more than one factor being eliminated [13]. Following the exploratory factor analysis, a scale composed of 15 items was obtained. The data identified four factors: Positive Emotional Regulation, explaining 15.24% of the variance; Compulsive Use, explaining 13.19% of the variance; Negative Emotional Regulation, explaining 12.58% of the variance; and Cognitive Preoccupation, explaining 8.20% of the variance.

The first factor, Positive Emotional Regulation, comprised four items reflecting the regulation of positive emotional states through the internet, such as “Whenever I am very happy, the first thing I do is use the internet to express it”. The second factor, Compulsive Use, consisted of four items reflecting difficulties in controlling and perceiving the time spent on the internet. For example, “I have difficulty controlling the amount of time I spend online”. The third factor, Negative Emotional Regulation, consisted of three items reflecting the use of the internet to alleviate negative moods. For example, “When I feel bad, I use the Internet to feel better”. Finally, the fourth factor, Cognitive Preoccupation, included four items reflecting a constant concern about connecting to the internet. For example, “I never usually worry about connecting to the internet”.

Factorial Validity of the Model

For the confirmatory factor analysis using maximum likelihood estimation, the same structure as Caplan’s original model (2010) [20] was proposed. The factors Positive Emotional Regulation and Negative Emotional Regulation were combined into a first-order latent factor called Emotional Regulation. The factors Compulsive Internet Use and Cognitive Preoccupation were grouped into another latent factor called Deficient Control (see Table 2, Model 1).

The resulting goodness-of-fit statistics values were acceptable after reaching the maximum number of iterations (see Table 2, Model 1). However, the regression weight between the Compulsive Use factor and Deficient Control was not significant (*p* = 0.911), leading to the rejection of Model 1). A second analysis was conducted by eliminating the Deficient Control latent factor. In this second analysis, all regression weights between the factors and items were significant (*p* < 0.001). The absolute value of Chi-square (X^2^) was 746.741, degrees of freedom (df) = 85, *p* < 0.05. The Normed Fit Index (NFI) showed a value of 0.95, and the Comparative Fit Index (CFI) had a value of 0.96. The Root Mean Square Error of Approximation (RMSEA) was 0.5, and the Standardized Root Mean-Square Residual (SRMR) was 0.39 (see Table 2, Model 2).

**Figure 1:**
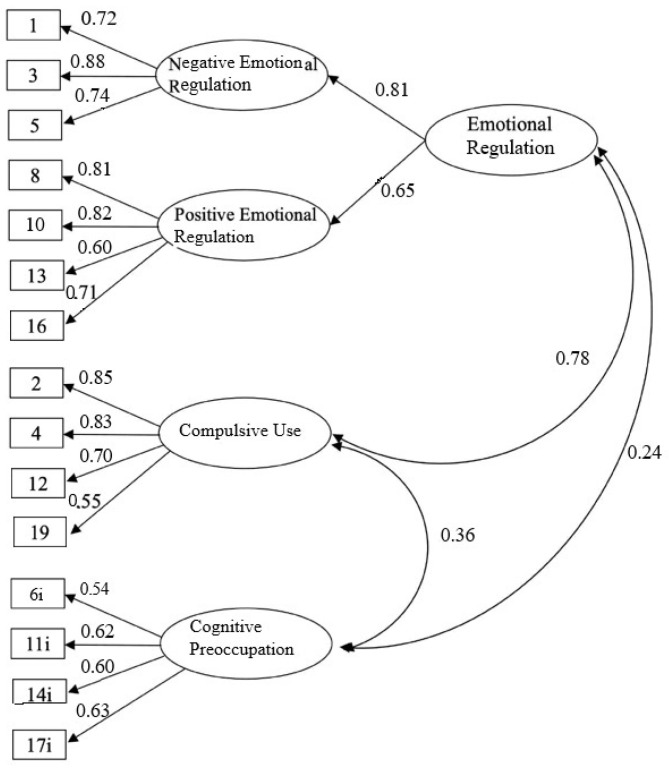
Schematic Representation of Confirmatory Factor Analysis of the measurement model. The numbers represent the covariances between the observable factors and the latent factor ((**right**), 0.24–0.78) and the factor loadings of the four factors ((**left**), 0.54–0.88). Emotional Regulation; Negative R.: Negative Emotional Regulation; Positive R.: Positive Emotional Regulation; Compulsive Use; Cognitive Preoccupation.

In Figure 1, the standardized factor loadings for each of the four factors in Model 2 are displayed. These loadings range from 0.54 to 0.88 (*p* < 0.001). Similarly, the values of the covariances between the factors Compulsive Use and Cognitive Preoccupation and the latent factor Emotional Regulation ranged from 0.24 to 0.78 (*p* < 0.001).

To determine reliability, a Cronbach’s Alpha internal consistency analysis was conducted. The results gathered in Table 3 show that there is good internal consistency for the factors of positive and negative emotional regulation (0.81–0.82). The cognitive worry factor exhibited acceptable internal consistency (0.69). In the case of the compulsive use factor, if the item “Right after finishing a meal or dinner, I connect to the internet” is removed, the internal consistency increases from 0.81 to 0.83.

Next, the association between the factors determined from Model 2 and the risk variables was determined. Pearson correlations were used. The correlation matrix was carried out between the four factors of Model 2 and the risk variables age, “Time Dedicated to the Internet”, “Social Networks” and “Instant Messaging”, the average of the grades obtained in natural sciences and social sciences. (see Table 4). The correlations of age and time spent on each application were positive and statistically significant, and negative and statistically significant with the average of the grades.

## 4. Discussion

The aim of this study was to design and validate an instrument capable of operationalizing a cognitive-behavioral model of maladaptive internet behavior. To achieve this, the model proposed by Caplan (2010) [20] was used as a reference, which explains Generalized Problematic Internet Use [1] represented by three factors: Preference for Online Social Interaction, Mood Regulation, and Lack of Control (comprising Compulsive Use and Cognitive Concern).

Our Exploratory Factor Analysis revealed that 15 out of the 19 designed items represent three of Caplan’s proposed factors (Negative Emotional Regulation, Compulsive Use, and Cognitive Concern), and a fourth factor, which we included in this study, Positive Emotional Regulation.

The Confirmatory Factor Analysis ruled out the second-order factor, Lack of Control, as it was not represented by the first-order Compulsive Use factor. The regression weight between the two was not significant (*p* = 0.911). This differs from Caplan’s model, which suggests that internet Lack of Control is composed of Compulsive Use and Cognitive Concern. This discrepancy between our data and Caplan’s proposal may be due to the different population samples used. While this study’s sample was adolescents (Mean: 13.55), Caplan’s sample consisted of adults (Mean: 33.14).

**Figure 2:**
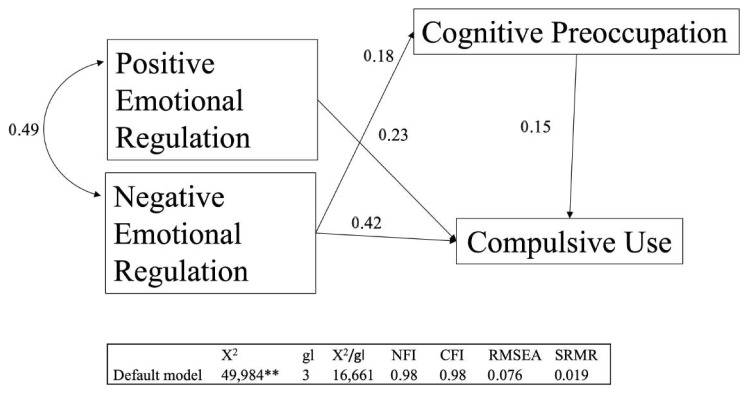
Schematic representation of the directions between the factors resulting from the Confirmatory Factor Analysis of Model 2. The numbers represent the factor loadings. The covariance between Positive Emotional Regulation and Negative Emotional Regulation was 0.49. Insert A: Goodness-of-fit statistics. X2, Chi-Square; df, degrees of freedom; NFI (Normed Fit Index); CFI (Robust Comparative Fit Index); RMSEA (Root Mean Square Error of Approximation); SRMR (Standardized Root Mean-square Residual). **: *p* ≤ 0.001.

In the second Confirmatory Factor Analysis (see Figure 2), the order of appearance of variables according to their influence on each other was determined. For example, in Caplan’s model, Negative Emotional Regulation has a direct influence on Lack of Control. Our data showed that Negative Emotional Regulation had a direct effect on Cognitive Concern and Compulsive Use. However, Positive Emotional Regulation only had an effect on Compulsive Use. The data from the second Confirmatory Factor Analysis presented values that were appropriate according to goodness-of-fit statistics. However, the RMSEA value of 0.086 is considered mediocre in an analysis of this nature, and therefore, all these paths were rejected [39,40]. Consequently, we decided to eliminate the direct effect of the Positive Emotional Regulation factor on Cognitive Concern because this factor was determined based on negative thoughts. Once this was completed, the RMSEA value showed an acceptable value of 0.076 [39,40]. The NFI and CFI values were 0.98 and 0.98, respectively. The SRMR value was close to zero (0.019).

Pearson correlation matrices show that the more dedication there is on workdays and weekends to internet browsing, social media use, and instant messaging, the more the behavior of Positive and Negative Emotional Regulation, Compulsive Use, and Cognitive Concern is exhibited. Time of use variables are one of the main risk factors for problematic use of information and communication technologies [10]. For example, in the Jo et al. study (2022) [32], using a sample of 644 adolescents, they reported that, on average, they played more than 1.5 h a day, and 64 of them showed problematic use. In Berchtold et al. study (2018) [41], the daily average internet use for a sample of 2942 adolescent subjects was 2.24 h. Internet use time increased with age between 13 and 15 years, and the increase in negative consequences, such as sleep problems, was greater as internet use time increased. In our study, the daily average use was 1 to 2 h on workdays and weekends, with higher use among females in social media and messaging every day (*p* < 0.001), and equal use between genders in internet browsing every day (*p* > 0.001). Furthermore, it is associated with greater maladaptive behavior as age increases in the range of 12 to 16 years and with academic performance.

Internal consistency analysis using reliability yielded acceptable reliability values, indicating a good representation of the items for each of the factors in line with the theoretical proposal. The internal consistency of the Compulsive Use factor significantly improved after removing one of the items.

### The Role of Positive Emotional Regulation in the Evaluation of PIU

The Positive Emotional Regulation has consistently been found to be involved in addiction to substance abuse [42] and behavioral addictions related to Internet use, including web browsing, video games, gambling with money, and social media [27,28,38,43]. Essentially, our proposal to include Positive Emotional Regulation in internet use is rooted in the very definition of Emotional Regulation, which is understood as a latent factor. Emotional Regulation has been defined as “the extrinsic and intrinsic processes responsible for monitoring, evaluating, and modifying emotional reactions, especially their intensity and temporal features, to accomplish one’s goals” (Thompson [44]). This definition aligns with the Positive Emotional Regulation factor, composed of the following four items from our questionnaire: “Whenever I am very happy, the first thing I do is use the internet to express it”, “When I am really content, I use the internet to communicate it”, “When I am in love, I always express it through the internet”, and “When I enjoy something, I use the internet to share it with others”. Therefore, despite Negative Emotional Regulation having a slightly higher weight (0.81) than Positive Emotional Regulation (0.65), according to our data, adding Positive Emotional Regulation would improve the screening of internet use disorders in the adolescent population.

Other theoretical models on problematic internet use support the need to introduce emotional regulation in assessments of adolescent populations. For example, the model proposed by Tunney and Rooney (2023) [10] suggests that variables involved in the process of problematic internet use indicate that the regulation of mood states can be considered a by-product of usage, leading to a habit and conditioning through which adolescents may become addicted to the internet. Our study not only supports the negative valence that has already been widely studied [2], but also the positive valence as a reinforcement strategy in situations or contexts perceived as pleasant. This can also help generate expectations of the internet approach, as indicated by the I-PACE model on internet addiction [21]. Finally, just as with the regulation of negative mood states, positive valence could act as a precipitating and maintaining factor, as indicated by Tunney and Rooney (2023) [10].

In our study, seven items make up the two Positive/Negative Emotional Regulation factors, in contrast to Caplan’s model, which consists of three items in a single Negative Emotional Regulation factor. This represents an advantage as it allows the evaluation of emotional regulation in two valences, negative and positive, understood as the emotional regulation of situations perceived as pleasant (positive emotional regulation) or unpleasant (negative emotional regulation) using the internet as a means to channel this emotion [10,30]. The consequence of this regulation ranges from positive reinforcement to maintain the pleasant state to negative reinforcement to alleviate the unpleasant state, that is, it is a balance of gratification and compensation that not only maintains behavior but also can be a precipitant [10,21]. When applied to clinical practice, this distinction allows for the determination of behavioral orientation and the setting of objectives, such as relaxation and cognitive restructuring for negative emotions and the control of excessive hours and social skills for positive emotions [45,46]. The other two factors, Compulsive Use and Cognitive Concern, each comprise four items. These factors are in line with Caplan’s model and represent variables that are closer to negative consequences, according to Caplan.

The highest-scoring item in each factor is as follows: “When I feel bad, I use the Internet to feel better” (Negative Emotional Regulation), “I usually don’t worry about connecting to the internet” (Cognitive Concern, reverse-scored item), “I am connected to the internet without realizing how much time passes” (Compulsive Use), and “When I enjoy something, I use the internet to share it with others” (Positive Emotional Regulation). These data align with the literature that shows the relationship between these psychological processes and with negative consequences of internet use in the general adolescent population [28,47,48].

Limitations encountered are seen in the study’s participants, as they are not clinical subjects. However, it is worth noting that there are currently no specific criteria for problematic internet use that are endorsed by major diagnostic manuals, such as DSM-5 or ICD-11. The cross-sectional nature of our study prevents the establishment of definitive causal relationships. Future longitudinal studies could address this limitation and offer a deeper understanding of the temporal dynamics between emotional regulation and internet use disorders. Furthermore, the generalizability of our findings might be limited by the specific sample used, suggesting the need to replicate the study in different populations and cultural contexts.

Another limitation was applying at the same time a test that evaluated a similar construct, that is, problematic Internet use. This would have allowed the test to be validated with a criterion external to the test, increasing the consistency of criterion validity, and ensuring that it measures the desired construct.

As future lines of research, it is necessary to evaluate these cognitive-behavioral processes regarding potential negative consequences not included in this study because we do not consider them as psychological processes involved in behavior, but as consequences derived from them. Furthermore, it is essential to explore whether the higher-level construct of Lack of Control [24] needs to be considered in the presence of negative consequences. On the other hand, this model should be evaluated in a clinical population.

## 5. Conclusions

We can conclude that the designed instrument is valid and reliable for identifying and discriminating adolescents with problematic internet use. The results shown in the Pearson correlations demonstrate that the processes studied are not only associated with mere internet use, understood as browsing, downloading files, or seeking information, but also with other applications, such as instant messaging and social media. Studies with these types of applications have shown this association between emotional regulation, compulsive use, and cognitive concern [7,14].

## Figures and Tables

**Table 1:** Resultant Factors from Exploratory Factor Analysis of Rotated Items. Rotated Factor Matrix.

Items	Positive Emotional Regulation	Compulsive Use	Negative Emotional Regulation	Cognitive Preoccupation
8	**0.773**	0.171	0.167	0.062
10	**0.778**	0.132	0.159	0.043
13	**0.556**	0.144	0.185	0.040
16	**0.658**	0.201	0.207	0.013
2	0.125	**0.804**	0.225	0.171
4	0.219	**0.722**	0.235	0.193
12	0.228	**0.636**	0.210	0.072
19	0.332	**0.425**	0.264	0.122
1	0.176	0.245	**0.632**	0.037
3	0.205	0.282	**0.774**	0.066
5	0.251	0.225	**0.704**	0.052
6	0.050	0.131	0.012	**0.502**
11	0.038	0.118	0.025	**0.601**
14	0.048	0.016	0.038	**0.625**
17	0.042	0.083	0.070	**0.628**
% of explained variance	15.24	13.19	12.58	8.20
KMO	0.9			

Note: The numbers in the table provide information about the internal structure of the test and the quality of the items in relation to the identified factors. The numbers in bold indicate that the item has a strong relationship with the factor in question, suggesting that the item is a good representative of the construct measured by that factor.

**Table 2:** Goodness-of-Fit Parameters for the Models.

	X^2^	df	X^2^/df	NFI	CFI	RMSEA	SRMR
**Model 1**							
Default model	750,039 *	85	8824	0.95	0.96	0.05	0.039
**Model 2**							
Default model	746,741 *	85	8785	0.95	0.96	0.05	0.039

Note: * *p* < 0.001. X^2^: Chi-square; df: degrees of freedom; NFI (Normed Fit Index); CFI (Robust Comparative Fit Index); RMSEA (Root Mean Square Error of Approximation); SRMR (Standardized Root Mean-square Residual).

**Table 3:** Reliability Analysis using Cronbach’s Alpha for Model 2.

	Alpha Cronbach if the Item Is Suppressed	Cronbach’s Alpha for the Factor
NEGATVE_R		
1	0.78	0.81
4	0.67	
7	0.78	
POSITIVE_R		
10	0.74	0.82
13	0.74	
16	0.82	
20	0.78	
Cognitive Preoccupation		
8i	0.65	0.69
14i	0.61	
17i	0.62	
21i	0.61	
COMPUL_USE		
3	0.72	0.81
6	0.74	
15	0.76	
24	0.83	

**Table 4:** Pearson Correlations between the Factors of Model 2 and risk variables.

	Negative R	Positive R	Compul_Use	Cog_Preocu
Age	0.130 **	0.076 **	0.126 **	0.059 **
Qualification in Natural Sciences (Biology, Physics, Mathematics, etc.)	−0.154 **	−0.179 **	−0.190 **	−0.116 **
Qualification in Social Sciences (Language, Literature, History)	−0.166 **	−0.196 **	−0.199 **	−0.132 **
In_Weekday	0.319 **	0.290 **	0.372 **	0.146 **
In_Weekend	0.314 **	0.270 **	0.352 **	0.167 **
Vg_Weekday	0.237 **	0.201 **	0.247 **	0.080 **
Vg_Weekend	0.173 **	0.100 **	0.174 **	0.081 **
Sm_Weekdays	0.328 **	0.404 **	0.395 **	0.201 **
Sm_Weekends	0.296 **	0.388 **	0.375 **	0.207 **
Im_Weekday	0.275 **	0.325 **	0.313 **	0.125 **
Im_Weekends	0.254 **	0.320 **	0.288 **	0.141 **

Age: age of the participants. Qualification in Natural Sciences (Biology, Physics, Mathematics, etc.): average grade in Natural Sciences. Qualification in Social Sciences (Language, Literature, History): average grade in social sciences. In_Weekday: Internet Weekday: Time spent browsing the internet from Monday to Friday. In_Weekend: Internet Weekend: Time spent browsing the internet on Saturday and Sunday. Vg_Weekday: Videogames Weekday: Time spent browsing videogames from Monday to Friday. Vg_Weekend: Videogame Weekend: Time spent browsing videogames on Saturday and Sunday. Sm_Weekdays: Social Media Weekday: Time spent on social media from Monday to Friday. Sm_Weekends: Social Media Weekend: Time spent on social media on Saturday and Sunday. Im_Weekday: Instant Messaging Weekday: Time spent on instant messaging from Monday to Friday. Im_Weekends: Instant Messaging Weekend: Time spent on instant messaging on Saturday and Sunday. Negative R.: Negative Emotional Regulation. Positive R.: Positive Emotional Regulation. Compul_Use: Compulsive Use. Cog_Preocu: Cognitive Preoccupation. Note ** *p* < 0.001.

## Data Availability

Data can be requested from the corresponding author.

## Supplementary Materials

The following supporting information can be downloaded at: https://www.mdpi.com/article/10.3390/bs14080715/s1, Table S1: Maladaptive behavior scale towards ICT (CD-TIC) (original—English version); Table S2: Escala de comportamiento desadaptativo hacia las TIC (CD-TIC) (original-Versión Española).
