# Supplementary material for: Validation of an Instrument for Detecting Problematic Internet Use in Adolescents
Source: Behav Sci (Basel). 2024 Aug 15;14(8):715. doi: 10.3390/bs14080715 (PMC11351187; doi:10.3390/bs14080715)
Supplement: Supplementary file 1 [file behavsci-14-00715-s001.zip › behavsci-3077876-supplementary.pdf]

Supplementary Material

Table S1. Maladaptive behavior scale towards ICT (CD-TIC) (original – English version)

1. When I feel bad, I use the Internet to feel better	1	2	3	4	5	6
2. I have difficulty controlling the amount of time I am connected to the Internet	1	2	3	4	5	6
3. When I am angry or upset about something, I use the internet to feel better.	1	2	3	4	5	6
4. I find it difficult to control my Internet use	1	2	3	4	5	6
5. To feel better I have used the Internet after a fight or argument	1	2	3	4	5	6
6. I never usually worry about connecting to the internet	1	2	3	4	5	6
7. The first thing I do when I wake up is quickly connect to the internet	1	2	3	4	5	6
8. Whenever I am very happy, the first thing I do is use the internet to show it.	1	2	3	4	5	6
9. Even if I'm busy with something, I can't resist looking at the internet from time to time.	1	2	3	4	5	6
10. When I am really happy I use the internet to communicate it	1	2	3	4	5	6
11. I find it easy not to worry about connecting to the internet	1	2	3	4	5	6
12. I am connected to the internet without realizing how much time passes	1	2	3	4	5	6
13. When I'm in love, I always express it through the internet	1	2	3	4	5	6
14. When I can't watch the internet for some reason, I don't worry about getting online.	1	2	3	4	5	6
15. When I argue with someone, the first thing I do is use the Internet to feel better.	1	2	3	4	5	6
16. When I have fun with something I use the internet to tell others about it.	1	2	3	4	5	6
17. If I lose my network, I don't worry about connecting to the internet.	1	2	3	4	5	6
18. Without fail every day before going to sleep I connect to the internet	1	2	3	4	5	6
19. As soon as I finish eating or dining I connect to the internet	1	2	3	4	5	6

Table S2. Escala de comportamiento desadaptativo hacia las TIC (CD-TIC) (original-Versión Española)

1. Cuando me siento mal, uso Internet para sentirme mejor	1	2	3	4	5	6
2. Tengo dificultad para controlar la cantidad de tiempo que estoy conectado a Internet	1	2	3	4	5	6
3. Cuando estoy enfadado o molesto por algo, uso internet para sentirme mejor	1	2	3	4	5	6
4. Me resulta difícil controlar mi uso de Internet	1	2	3	4	5	6
5. Para sentirme mejor he usado Internet tras una pelea o discusión	1	2	3	4	5	6
6. Nunca suelo preocuparme por conectarme a internet	1	2	3	4	5	6
7. Lo primero que hago al despertarme es conectarme rápidamente a internet	1	2	3	4	5	6
8. Siempre que estoy muy feliz lo primero que hago es utilizar internet para mostrarlo	1	2	3	4	5	6
9. Aunque esté entretenido con algo, no me resisto a mirar internet de vez en cuando	1	2	3	4	5	6
10. Cuando estoy realmente contento uso internet para comunicarlo	1	2	3	4	5	6
11. Me resulta fácil no preocuparme en conectarme a internet	1	2	3	4	5	6
12. Estoy conectado a internet sin darme cuenta del tiempo que pasa	1	2	3	4	5	6
13. Cuando estoy enamorado, siempre lo expreso a través de internet	1	2	3	4	5	6
14. Cuando no puedo mirar internet por algún motivo, no me preocupo por conectarme	1	2	3	4	5	6
15. Cunado discuto con alguien lo primero que hago es utilizar internet para sentirme mejor	1	2	3	4	5	6
16. Cuando me divierto con algo utilizo internet para contárselo a los demás	1	2	3	4	5	6
17. Si me quedo sin red no me preocupo en conectarme a internet	1	2	3	4	5	6
18. Sin falta todos los días antes de dormir me conecto a internet	1	2	3	4	5	6
19. Nada más terminar de comer o cenar me conecto a internet	1	2	3	4	5	6
